# Bacterial communities on classroom surfaces vary with human contact

**DOI:** 10.1186/2049-2618-2-7

**Published:** 2014-03-07

**Authors:** James F Meadow, Adam E Altrichter, Steven W Kembel, Maxwell Moriyama, Timothy K O’Connor, Ann M Womack, G Z Brown, Jessica L Green, Brendan J M Bohannan

**Affiliations:** 1 Biology and the Built Environment Center, Institute of Ecology and Evolution, University of Oregon, 5389 University of Oregon, Eugene, OR 97403, USA; 2 Department of Biological Sciences, University of Quebec, 320 Rue Sainte-Catherine Est, Montréal, QC H2X 1 L7, Canada; 3 Energy Studies in Buildings Laboratory, Department of Architecture, University of Oregon, 1206 University of Oregon, Eugene, OR 97403, USA; 4 Department of Ecology and Evolutionary Biology, University of Arizona, BioSciences West room 310, 1041 E. Lowell St, Tucson, AZ 85721, USA; 5 Santa Fe Institute, 1399 Hyde Park Rd, Santa Fe, NM 87501, USA

**Keywords:** Built environment, Microbial ecology, Indoor microbiology, *Lactobacillus*

## Abstract

**Background:**

Humans can spend the majority of their time indoors, but little is known about the interactions between the human and built-environment microbiomes or the forces that drive microbial community assembly in the built environment. We sampled 16S rRNA genes from four different surface types throughout a university classroom to determine whether bacterial assemblages on each surface were best predicted by routine human interactions or by proximity to other surfaces within the classroom. We then analyzed our data with publicly-available datasets representing potential source environments.

**Results:**

Bacterial assemblages from the four surface types, as well as individual taxa, were indicative of different source pools related to the type of human contact each surface routinely encounters. Spatial proximity to other surfaces in the classroom did not predict community composition.

**Conclusions:**

Our results indicate that human-associated microbial communities can be transferred to indoor surfaces following contact, and that such transmission is possible even when contact is indirect, but that proximity to other surfaces in the classroom does not influence community composition.

## Background

In the developed world, humans spend a majority of their lives indoors. While indoors we encounter microorganisms on virtually every surface we touch, and this frequent exposure to indoor microbes carries with it the potential for disease transmission, as well as interactions with our own commensal microbiome [1-3]. Yet we have very little knowledge regarding the ecological processes that drive microbial community assembly indoors, nor do we understand the degree to which humans share microbes with indoor surfaces.

For any given indoor surface, microbial community composition is likely shaped by habitat-specific environmental constraints (such as the type of surface material), and dispersal sources (which include humans, bioaerosols and other surfaces within a space). If dispersal among surfaces is a primary determinant of community structure, then adjacent surfaces should be more similar in community composition than surfaces further apart. If dispersal from humans is a major determinant, then community structure should vary with frequency and nature of human contact.

Because humans harbor distinguishably different microbial assemblages on different parts of their bodies [4-6], it is reasonable to assume that different indoor surface types could harbor different microbial communities due to frequent contact with specific body parts. Several studies suggest that this may be the case. In a recent survey of public restroom surfaces, Flores and colleagues [7] found that microbes on restroom surfaces were similar to those found on specific human body parts, with the strongest association observed between toilet surfaces and gut and vaginal communities. These associations are to be expected given the direct contact common in this environment between surfaces and the human body. Associations between human use and bacterial community composition have also been found on residential kitchen surfaces [8], with bacterial taxa commonly found on human skin predominating on kitchen surfaces, consistent with frequent skin-to-surface contact. It is not known whether more indirect skin-to-surface contact, such as sitting fully clothed on a chair, might result in similar bacterial transmission.

Here we test: (a) if different types of surfaces in a classroom vary in microbial community composition; (b) if their composition varies predictably with the type of human contact; and (c) whether these associations are stronger than the association between community similarity and spatial distance. We sampled desktops, floors, walls and chair seats in a classroom to determine whether these groups of surfaces harbored distinct bacterial assemblages related to their differing types of human contact.

## Methods

**Figure 1:**
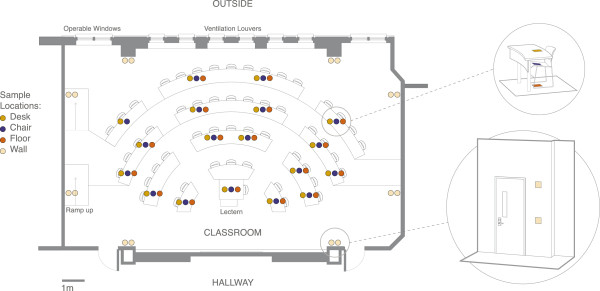
**Schematic of sampling design.** Four different types of surfaces (desks, chairs, floors and walls) were sampled throughout an amphitheater-style classroom.

All samples were collected in the same classroom at the Lillis Business Complex, University of Oregon, Eugene, OR, USA, during 4–5 August 2011. During the week prior to the sampling period, the classroom was occupied by classes daily, including on the first day of sampling. Desks, floors, chair seats and walls were sampled (*n* = 18, 18, 18 and 16, respectively) following identical protocols. Samples were distributed throughout the classroom (9.2 m × 15.2 m). Pairwise spatial distance between samples ranged from 0 to 16 m, with sets of chair, desk and floor samples generally taken within 1 m^2^ of one another. Figure 1 displays the spatial distribution of samples within the classroom. Surfaces were sampled using a nylon flocked swab (copanusa.com; #552C) moistened with sterile buffer solution (0.15 M NaCl, 0.1 Tween20). A 289 cm^2^ (17 cm × 17 cm) area was swabbed from each surface. Chairs were sampled in the center of their upholstered seat surfaces; desks (plastic laminate surface) and floors (linoleum) were sampled directly above and below chair seats; and walls (latex paint) were sampled around the perimeter of the room (Figure 1). We were not able to ascertain the cleaning schedule prior to sampling, but all surfaces were visibly devoid of standing dust. All samples were frozen (−80°C) until DNA extraction.

DNA extraction, amplification and Illumina library preparation followed methods described previously [9,10]. DNA was extracted from swabs using a PowerWater DNA extraction kit (MoBio Laboratories, Inc., Carlsbad, CA, USA) with the following modifications: samples were frozen and thawed for two cycles; bead beating length was extended to 10 minutes; and samples were eluted in 50 μL Solution PW6.

The V4 region of the 16S rRNA gene was amplified using the F515/R806 primer combination (5′-GTGCCAGCMGCCGCGG-3′, 5′-TACNVGGGTATCTAATCC-3′; [11]). Amplification proceeded in two steps using a custom Illumina preparation protocol where PCR1 was performed with forward primers that contained partial unique barcodes and partial Illumina adapters. The remaining ends of the Illumina adapters were attached during PCR2, and barcodes were recombined *in silico* using paired-end reads. Adapter sequences are detailed in Additional file 1. All extracted samples were amplified in triplicate for PCR1 and triplicates were pooled before PCR2. PCR1 (25 μL total volume per reaction) consisted of the following steps: 5 μL 5× HF buffer (Thermo Fisher Scientific, Waltham, MA, USA), 0.5 μL deoxyribonucleotide triphosphates (10 mM, Invitrogen, Life Technologies, Grand Island, NY, USA), 0.25 μL Phusion Hotstart II polymerase (0.5 units; Thermo Fisher Scientific), 13.25 μL certified nucleic-acid free water, 0.5 μL (10 μM) forward primer, 0.5 μL (10 μM) reverse primer, and 5 μL template DNA. The PCR1 conditions were as follows: initial denaturation for 2 minutes at 98°C; 22 cycles of 20 seconds at 98°C, 30 seconds at 50°C and 20 seconds at 72°C; and 72°C for 2 minutes for final extension. After PCR1, the triplicate reactions were pooled and cleaned with the Qiagen MinElute PCR Purification Kit according to the manufacturer’s protocol (Qiagen, Germantown, MD, USA). Samples were eluted in 11.5 μL Buffer EB (10 mM Tris-Cl, pH 8.5, Qiagen). For PCR2, a single primer pair was used to add the remaining Illumina adapter segments to the ends of the concentrated amplicons of PCR1. The PCR2 (25 μL volume per reaction) consisted of the same combination of reagents that was used in PCR1, along with 5 μL concentrated PCR1 product as a template. The PCR2 conditions were as follows: 2 minutes denaturation at 98°C; 12 cycles of 20 seconds at 98°C, 30 seconds at 66°C and 20 seconds at 72°C; and 2 minutes at 72°C for final extension.

Amplicons were size selected by gel electrophoresis, extracted, quantified on a Qubit 2.0 Fluorometer (Life Technologies, Carlsbad, CA, USA), concentrated, combined in equimolar concentrations and sequenced on the Illumina MiSeq platform at the Dana-Farber/Harvard Cancer Center DNA Resource Core (Boston, MA; dnaseq.med.harvard.edu).

Sequence processing was performed using the FastX Toolkit (http://hannonlab.cshl.edu/fastx_toolkit) and QIIME [12]. Quality filtering settings were as follows: a minimum 30 quality score over at least 75% of the sequence read, no ambiguous bases, and 1 primer mismatch allowed. After quality control and barcode assignment, the remaining high-quality sequences (mean 13,786 sequences per sample ± 4,735 SD) were binned into operational taxonomic units (OTUs) at a 97% sequence similarity cutoff using uclust [13]. High-quality sequences from each OTU cluster were taxonomically identified using reference sequences from the Greengenes database (2011 release; [14,15]), and plant-chloroplast OTUs were removed by name (“Streptophyta”) based on Greengenes taxonomic classifications (mean = 8% of sequences in each sample ± 10% SD). All samples retaining more than 4,000 sequences (*n* = 15, 14, 15 and 14 for desks, walls, floors, and chairs, respectively) were rarefied to that level for even sampling depth. Sequence files and metadata for all samples used in this study have been deposited in Figshare (http://dx.doi.org/10.6084/m9.figshare.687155). Metadata, the unrarefied OTU table, and corresponding taxonomic classifications have all been included as Additional files 2, 3 and 4, respectively.

All statistical analyses were performed in R [16], primarily utilizing multivariate community ecology procedures in the labdsv and vegan packages [17,18]. A full record of all statistical analysis is included as Additional file 5, and was created using the knitr package in R [19]. Community distance between samples was calculated using the Canberra distance, implemented in the vegan package. Given that the four surface types we sampled represent potentially similar microbial habitats, we did not expect to see major phylogenetic differences among the communities. Thus, we used the Canberra taxonomic metric as a way to emphasize community differences driven by relatively rare taxa with a decreased emphasis on total abundance and phylogenetic community distance. Distance-based redundancy analysis (DB-RDA) was performed with the capscale function in vegan. Individual OTUs exert varying degrees of influence, or weight, on DB-RDA clustering patterns, and we selectively tested the strength of association between the strongest weighted individual bacterial OTUs and relative surface types (that is, indicator value, as defined in [20]) with the indval function in labdsv. Mantel tests, using the mantel function in vegan, were used to test for a community dissimilarity correlation with spatial distance within the classroom for each of the four surface types, as well as all sites regardless of surface type. To assess community similarity to various potential source habitats (soil, aquatic, phyllosphere, human skin and human gut), we compared all samples at the class level to publicly-available datasets from representative potential source environments [4,21-24] using principal components analysis. Positions of each point in relation to source environments along the first principal component are used to compare their similarity to either human skin or phyllosphere bacterial communities.

## Results and discussion

**Figure 2:**
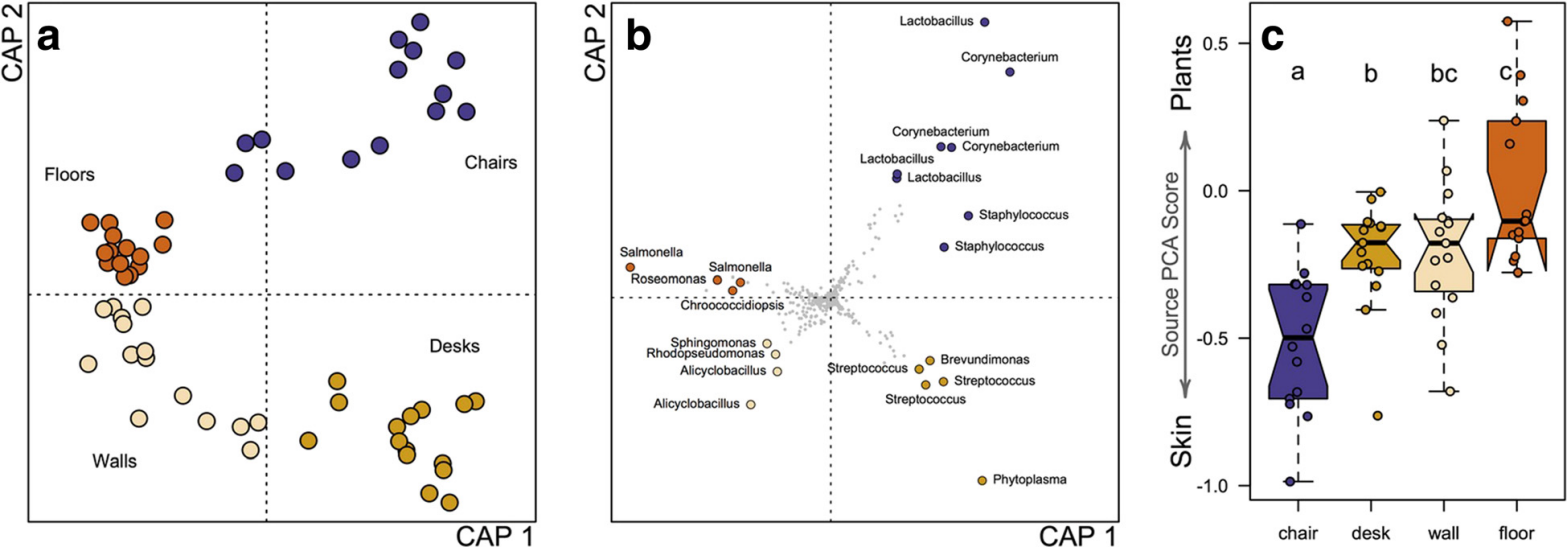
**Surfaces harbored significantly different bacterial communities and were linked to differential human contact. (a)** Bacterial communities were constrained by four different surface types using distance-based redundancy analysis (DB-RDA; constrained inertia = 11.4%) and were significantly different among types based on Canberra taxonomic distances (*P* = 0.001 from permutational multivariate analysis of variation). **(b)** Bacterial operational taxonomic units (OTUs) from DB-RDA are shown weighting communities in the same four primary directions. The first and second axes from DB-RDA are used in both ordinations (CAP 1 and CAP 2) . The strongest ten weighting OTUs for each surface type are highlighted if they were also significant indicator OTUs (all *P* values <0.05). **(c)** All samples were compared to potential source environments using principal components analysis (PCA), and the first principal component (37.8% of variance explained) was used as a surrogate for community similarity to either phyllosphere or human skin bacterial communities. Boxplots delineate (from bottom) minimum value, Q1, median (Q2), Q3, maximum value; notches approximate 95% confidence around median value, and outliers fall outside of the quartile range. Letters above each box indicate significant groupings after Tukey’s hones significant difference (HSD) test (adjusted *P* value <0.05).

After rarefaction to 4,000 sequences per sample, 58 surface samples were represented by a total of 3,745 bacterial OTUs. Approximately half (51.4%) of OTUs were found only once or twice, and these were included in analysis. The most commonly detected OTU (*Sphingomonas* sp.) accounted for 1.9% of all sequences. The four surface types harbored significantly different communities (*P* = 0.001; F = 2.34; from permutational multivariate analysis of variation on Canberra distances; Figure 2). Within each surface type, bacterial community composition was not significantly predicted by spatial distance among samples (all four *P* values >0.1; from Mantel tests). Even when all surfaces were considered together, spatial distance was not a significant predictor of bacterial community composition (*P* = 0.4). These results suggest that site-specific factors (for example, habitat selection or dispersal from specific body sites) are more important than dispersal among sites for bacterial community assembly in the built environment.

**Figure 3:**
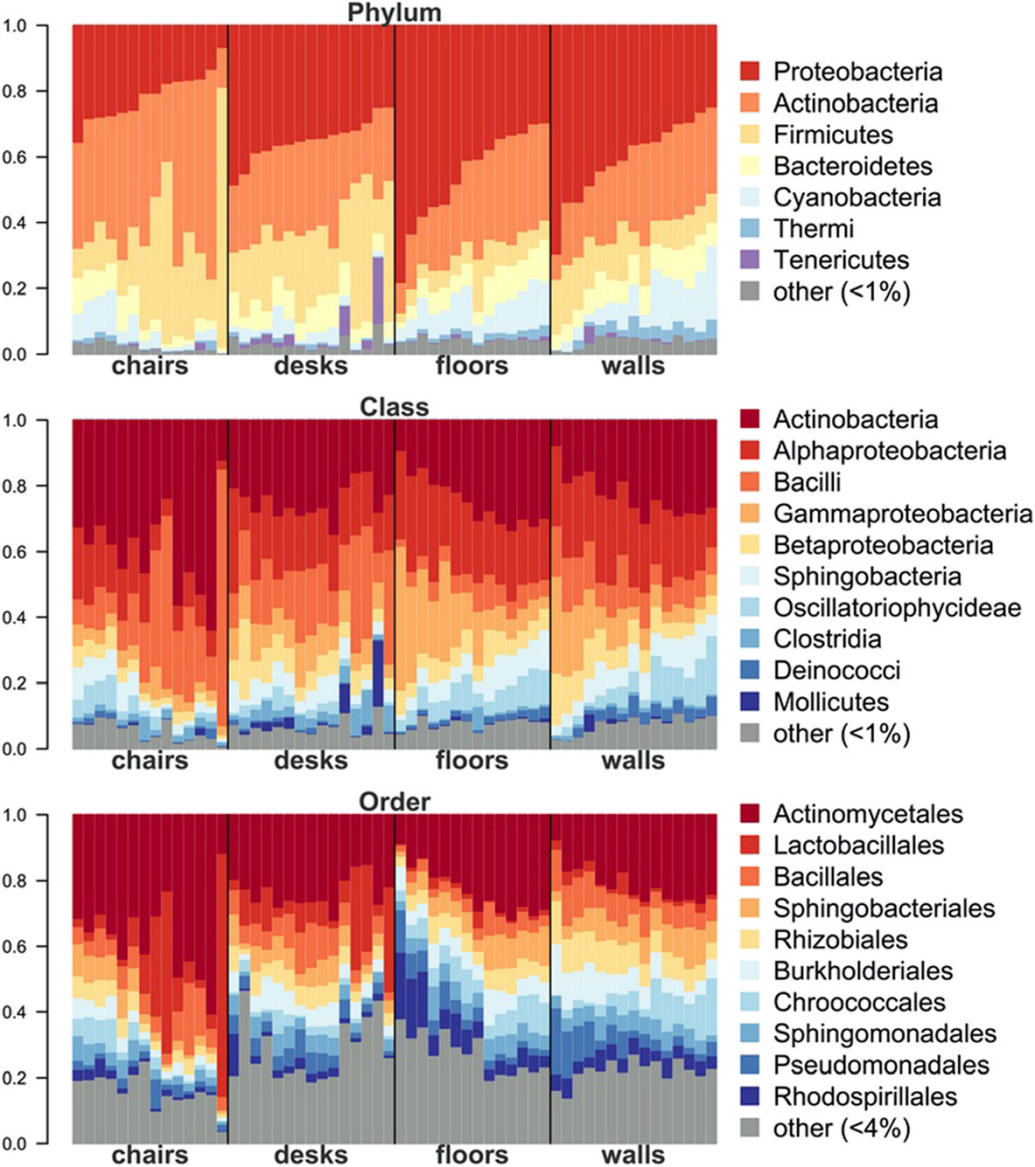
**Taxonomic composition of all 58 samples used in this study.** Samples are grouped by surface type. All taxonomic groups representing <1% (Phylum and Class) and <4% (Order) of sequences were grouped into ‘other’.

We found that surface communities were significantly associated with taxa specific to distinct microbial sources (Table 1). Bacterial OTUs labeled in Figure 2b are the most strongly weighted OTUs for each surface type that are also significant (*P* < 0.05) indicator OTUs. Three of the strongest indicators for chair seats were *Lactobacillus* OTUs closely related to taxa commonly found in the human gut and vagina, in addition to other human-associated *Corynebacterium* and *Staphylococcus* species. Desk surfaces were significantly associated with several indicator taxa from human body habitats; two of the strongest desk surface indicator OTUs were *Streptococcus* species commonly found in human skin and oral samples, as well as another *Streptococcus* originally isolated from purulent infections. Floors in the classroom also harbored skin-associated OTUs, but were more strongly indicated by a cyanobacterial OTU, presumably from a non-human environmental source. Walls are likely the surfaces in this study that have the least contact with humans; this was consistent with our observation that these surfaces were associated with indicator taxa related to *Sphingomonas* and *Alicyclobacillus* species that are commonly abundant in airborne bacterial assemblages [10,25]. Both walls and floors held larger relative proportions of Cyanobacteria than seats and desktops, likely reflecting soil and bioaerosols as microbial sources. Few other major phylogenetic differences are evident at a broad taxonomic level (Figure 3).

**Table 1:** Closest known isolates related to indicator operational taxonomic units

**Greengenes genus**	***P *** **value**	**Surface type**	**Closest 16S NCBI isolate and accession**	**Isolate source environment**	**Sequence similarity to isolate (%)**
*Lactobacillus*	0.001*	Chairs	*Lactobacillus johnsonii* NR_075064.1	Human gut	99
*Corynebacterium*	0.001*	Chairs	*Corynebacterium resistens* NR_040999.1	Human infection	99
*Corynebacterium*	0.001*	Chairs	*Corynebacterium confusum* NR_026449.1	Human clinical specimens	99
*Staphylococcus*	0.011*	Chairs	*Staphylococcus epidermidis* NR_074995.1	Human skin	99
*Corynebacterium*	0.001*	Chairs	*Corynebacterium riegelii* NR_026434.1	Human urinary tract	99
*Staphylococcus*	0.019*	Chairs	*Staphylococcus saprophyticus* NR_074999.1	Human urinary tract	99
*Lactobacillus*	0.001*	Chairs	*Lactobacillus crispatus* NR_074986.1	Human vagina	99
*Lactobacillus*	0.003*	Chairs	*Lactobacillus acidophilus* NR_075049.1	Human gut	99
*Streptococcus*	0.001*	Desks	*Streptococcus oralis* NR_102809.1	Human oral	99
*Streptococcus*	0.001*	Desks	*Streptococcus salivarius* NR_102816.1	Human oral	99
*Brevundimonas*	0.002*	Desks	*Brevundimonas variabilis* NR_037106.1	Pond water	99
*Streptococcus*	0.001*	Desks	*Streptococcus intermedius* NR_102797.1	Human purulent infection	99
*CandidatusPhytoplasma*	0.001*	Desks	None**	-	-
*Alicyclobacillus*	0.001*	Walls	*Tumebacillus permanentifrigoris* NR_043849.1	Soil	99
*Chroococcidiopsis*	0.028*	Walls	*Halospirulina tapeticola* NR_026510.1	Saline aquatic	96
*Alicyclobacillus*	0.001*	Walls	*Tumebacillus permanentifrigoris* NR_043849.1	Soil	98
*Rhodopseudomonas*	0.001*	Walls	*Methylobacterium adhaesivum* NR_042409	Drinking water	98
*Salmonella*	0.001*	Floors	*Pantoea ananatis* NR_103927.1	Phyllosphere	99
*Roseomonas*	0.001*	Floors	*Roseomonas gilardii* NR_029061.1	Human blood	99
*Roseomonas*	0.001*	Floors	*Roseomonas frigidaquae* NR_044455.1	Water-cooling system	99
*Salmonella*	0.001*	Floors	*Pantoea ananatis* NR_103927.1	Phyllosphere	99

All extant operational taxonomic units labeled in Figure 2 (and thus influential in distance-based redundancy analysis, as well as significant indicator taxa for their respective surface type) were related to their closest known bacterial isolate using 16S rRNA sequences in the NCBI Bacteria & Archaea Isolate Database. Source environments are from each isolate’s respective published source environment. *Unadjusted *P* value < 0.05. **Closest known isolate 89% similar. NCBI: National Center for Biotechnology Information.

The community variation we observed among surface types could be the result of either surface-specific environmental filtering or dispersal. Evidence to date suggests that microbial communities on indoor surfaces, with a few exceptions, are likely primarily dispersal-driven given the well-documented inputs from dust, settled airborne particles, outdoor air and surface contact [7-9,25-27]. The relative importance of in situ community dynamics and habitat filtering remain to be fully understood in the built environment.

Dispersal between human skin and contacted surfaces is well documented, primarily in a medical context using known pathogenic strains [1,28,29]. However, the vast majority of bacteria on and in the human body are not pathogens, but rather appear to be commensal components of our own microbiome [30]. Recent evidence has suggested that the unique microbial assemblages detected on different human body parts can be transferred to indoor surfaces following contact [26,31], and that these bacterial traces of human contact are evident in places such as restrooms [7]. Our results suggest that this transmission is possible even when that contact is indirect, and that such contact has a greater impact on microbial community structure indoors than dispersal among surfaces.

## Conclusion

We are constantly surrounded by diverse microbial communities indoors, and we are just beginning to understand how our interactions in the built environment shape those communities and our own human microbiome. Our results indicate that human-associated microbial communities can be transferred to indoor surfaces following contact, and that such transmission is possible even when contact is indirect, but that proximity to other surfaces in the classroom does not influence community composition.

### Availability of supporting data

Sequence files and metadata for all samples used in this study have been deposited in Figshare (http://dx.doi.org/10.6084/m9.figshare.687155). A full record of all statistical analysis is included as Additional file 5, and was created using the knitr package in R [19]. Original R scripts are available in GitHub (https://github.com/jfmeadow/Meadow_etal_Surfaces). Metadata, the unrarefied OTU table, and corresponding taxonomic classifications have all been included as Additional files 2, 3 and 4, respectively.

## Abbreviations

DB-RBA: distance-based redundancy analysis; OTU: operational taxonomic unit; PCR: polymerase chain reaction.

## Competing interests

The authors declare that they have no competing interests.

## Authors’ contributions

JFM analyzed the data and wrote the manuscript. AEA performed laboratory assays. SWK conceived the study and collected samples. MM conceived the study, prepared figures and collected samples. TKO conceived the study, performed laboratory assays and collected samples. AMW conceived the study and collected samples. GZB contributed resources. JLG conceived the study, contributed resources and collected samples. BJMB conceived the study, contributed resources and collected samples. All authors edited the manuscript and approved the final draft.

## Supplementary Material

Additional file 1 Explanation of partial adapter sequences used in Illumina amplicon library preparation. Click here for file

Additional file 2 Metadata associated with all samples used in this study. Click here for file

Additional file 3 Unrarefied operational taxonomic unit table used for this study. Click here for file

Additional file 4 **Taxonomic assignments for all operational taxonomic units in Additional file****3****.** Click here for file

Additional file 5 Full account of statistical analysis performed in R. Click here for file

## References

[B1] Pessoa-Silva CL Dharan S Hugonnet S Touveneau S Posfay-Barbe K Pfister R Pittet D Dynamics of bacterial hand contamination during routine neonatal care Infect Control Hosp Epidemiol 2004 25 192 197 10.1086/502376 15061408

[B2] Fujimura KE Demoor T Rauch M Faruqi AA Jang S Johnson CC Boushey HA Zoratti E Ownby D Lukacs NW 2013 House dust exposure mediates gut microbiome Lactobacillus enrichment and airway immune defense against allergens and virus infection. Proc Natl Acad Sci: Lynch SV doi:10.1073/pnas.1310750111 10.1073/pnas.1310750111 PMC3896155 24344318

[B3] Davis MF Iverson SA Baron P Vasse A Silbergeld EK Lautenbach E Morris DO Household transmission of meticillin-resistant Staphylococcus aureus and other staphylococci Lancet Infect Dis 2012 12 703 716 10.1016/S1473-3099(12)70156-1 22917102

[B4] Costello EK Lauber CL Hamady M Fierer N Gordon JI Knight R Bacterial community variation in human body habitats across space and time Science 2009 326 1694 1697 10.1126/science.1177486 19892944 PMC3602444

[B5] Fierer N Hamady M Lauber CL Knight R The influence of sex, handedness, and washing on the diversity of hand surface bacteria Proc Natl Acad Sci USA 2008 105 17994 17999 10.1073/pnas.0807920105 19004758 PMC2584711

[B6] Grice EA Kong HH Conlan S Deming CB Davis J Young AC Bouffard GG Blakesley RW Murray PR Green ED Turner ML Segre JA Topographical and temporal diversity of the human skin microbiome Science 2009 324 1190 1192 10.1126/science.1171700 19478181 PMC2805064

[B7] Flores GE Bates ST Knights D Lauber CL Stombaugh J Knight R Fierer N Microbial biogeography of public restroom surfaces PLoS One 2011 6 e28132 10.1371/journal.pone.0028132 22132229 PMC3223236

[B8] Flores GE Bates ST Caporaso JG Lauber CL Leff JW Knight R Fierer N Diversity, distribution and sources of bacteria in residential kitchens Environ Microbiol 2013 15 588 596 10.1111/1462-2920.12036 23171378 PMC5100818

[B9] Meadow JF Bateman AC Herkert KM O’Connor TK Green JL Significant changes in the skin microbiome mediated by the sport of roller derby PeerJ 2013 1 e53 23638391 10.7717/peerj.53 PMC3628844

[B10] Meadow JF Altrichter AE Kembel SW Kline J Mhuireach G Moriyama M Northcutt D O’Connor TK Womack AM Brown GZ Green JL Bohannan BJM Indoor airborne bacterial communities are influenced by ventilation, occupancy, and outdoor air source Indoor Air 2013 24 41 48 23621155 10.1111/ina.12047 PMC4285785

[B11] Liu Z Lozupone CA Hamady M Bushman FD Knight R Short pyrosequencing reads suffice for accurate microbial community analysis Nucleic Acids Res 2007 35 e120 10.1093/nar/gkm541 17881377 PMC2094085

[B12] Caporaso JG Kuczynski J Stombaugh J Bittinger K Bushman FD Costello EK Fierer N Pena AG Goodrich JK Gordon JI Huttley GA Kelley ST Knights D Koenig JE Ley RE Lozupone CA McDonald D Muegge BD Pirrung M Reeder J Sevinsky JR Tumbaugh PJ Walters WA Widmann J Yatsunenko T Zaneveld J Knight R QIIME allows analysis of high-throughput community sequencing data Nat Methods 2010 7 335 336 10.1038/nmeth.f.303 20383131 PMC3156573

[B13] Edgar RC Search and clustering orders of magnitude faster than BLAST Bioinformatics 2010 26 2460 2461 10.1093/bioinformatics/btq461 20709691

[B14] DeSantis TZ Hugenholtz P Larsen N Rojas M Brodie EL Keller K Huber T Dalevi D Hu P Andersen GL Greengenes, a chimera-checked 16S rRNA gene database and workbench compatible with ARB Appl Environ Microbiol 2006 72 5069 5072 10.1128/AEM.03006-05 16820507 PMC1489311

[B15] Cole JR Wang Q Cardenas E Fish J Chai B Farris RJ Kulam-Syed-Mohideen AS McGarrell DM Marsh T Garrity GM Tiedje JM The Ribosomal Database Project: improved alignments and new tools for rRNA analysis Nucleic Acids Res 2009 37 141 145 10.1093/nar/gkn879 PMC2686447 19004872

[B16] R Development Core Team R: A Language and Environment for Statistical Computing 2010 [http://cran.r-project.org]

[B17] Roberts DWlabdsv 2010 Ordination and Multivariate Analysis for Ecology [http://cran.r-project.org/web/packages/labdsv/index.html]

[B18] Oksanen J Blanchet FG Kindt R Legendre P O’Hara RB Simpson GL Solymos P Stevens MHH Wagner Hvegan 2011 Community Ecology Package [http://cran.r-project.org/web/packages/vegan/index.html]

[B19] Xie Y knitr: a comprehensive tool for reproducible research in R In Implementing Reprodcible Compututational Research 2013 Chapman and Hall/CRC: Stodden V, Leisch F, Peng RD

[B20] Dufrene M Legendre P Species assemblages and indicator species: the need for a flexible asymmetrical approach Ecol Monogr 1997 67 345 366

[B21] Redford AJ Fierer N Bacterial succession on the leaf surface: a novel system for studying successional dynamics Microb Ecol 2009 58 189 198 10.1007/s00248-009-9495-y 19221834

[B22] Nemergut DR Townsend AR Sattin SR Freeman KR Fierer N Neff JC Bowman WD Schadt CW Weintraub MN Schmidt SK The effects of chronic nitrogen fertilization on alpine tundra soil microbial communities: implications for carbon and nitrogen cycling Environ Microbiol 2008 10 3093 3105 10.1111/j.1462-2920.2008.01735.x 18764871

[B23] Hansel CM Fendorf S Jardine PM Francis CA Changes in bacterial and archaeal community structure and functional diversity along a geochemically variable soil profile Appl Environ Microbiol 2008 74 1620 1633 10.1128/AEM.01787-07 18192411 PMC2258623

[B24] Crump BC Peterson BJ Raymond PA Amon RMW Rinehart A McClelland JW Holmes RM Circumpolar synchrony in big river bacterioplankton Proc Natl Acad Sci USA 2009 106 21208 21212 10.1073/pnas.0906149106 19940248 PMC2783008

[B25] Qian J Hospodsky D Yamamoto N Nazaroff WW Peccia J Size-resolved emission rates of airborne bacteria and fungi in an occupied classroom Indoor Air 2012 22 339 351 10.1111/j.1600-0668.2012.00769.x 22257156 PMC3437488

[B26] Fierer N Lauber CL Zhou N McDonald D Costello EK Knight R Forensic identification using skin bacterial communities Proc Natl Acad Sci USA 2010 107 6477 6481 10.1073/pnas.1000162107 20231444 PMC2852011

[B27] Kembel SW Meadow JF O’Connor TK Mhuireach G Northcutt D Kline J Moriyama M Brown GZ Bohannan BJM Green JL Architectural design drives the biogeography of indoor bacterial communities PLoS One 2014 9 e87093 10.1371/journal.pone.0087093 24489843 PMC3906134

[B28] Noble WC Habbema JDF van Furth R Smith I de Raay C Quantitative studies on the dispersal of skin bacteria into the air J Med Microbiol 1976 9 53 61 10.1099/00222615-9-1-53 1263248

[B29] Pittet D Dharan S Touveneau S Sauvan V Perneger TV Bacterial contamination of the hands of hospital staff during routine patient care Arch Intern Med 1999 159 821 826 10.1001/archinte.159.8.821 10219927

[B30] The Human Microbiome Project Consortium Structure, function and diversity of the healthy human microbiome Nature 2012 486 207 214 10.1038/nature11234 22699609 PMC3564958

[B31] Hübner N-O Hübner C Kramer A Assadian O Survival of bacterial pathogens on paper and bacterial retrieval from paper to hands: preliminary results Am J Nurs 2011 111 30 34 22082498 10.1097/01.NAJ.0000408181.37017.82

