# Supplementary material for: Bacterial communities on classroom surfaces vary with human contact
Source: Microbiome. 2014 Mar 7;2:7. doi: 10.1186/2049-2618-2-7 (PMC3945812; doi:10.1186/2049-2618-2-7)

Bacterial community analysis for surfaces in a university classroom

Meadow *et al.* "Bacterial communities on classroom surfaces vary with human contact"

This document contains all statistical analyses conducted for the manuscript. Note that due to the random iterative nature of some analyses (such as beta-rarefaction, DB-RDA & PCA) some of the figure parameters will change slightly during reanalysis, though core results will remain essentially unchanged.

All data to reproduce analysis can be found here:
`https://github.com/jfmeadow/Meadow_etal_Surfaces`

Load necessary ecological analysis libraries.

```
library(vegan)
```

```
## Loading required package: permute This is vegan 2.0-8
```

```
library(labdsv)
```

```
## Loading required package: mgcv This is mgcv 1.7-22. For overview
type
## 'help("mgcv-package")'. Loading required package: MASS
##
## Attaching package: 'labdsv'
##
## The following object is masked from 'package:stats':
##
## density
```

Pull workspace from big OTU table created for Lillis Air data (Meadow et al. 2013, Indoor Air). Also removed a list of plants and 'no blast hits' as described in that manuscript.

This beginning workspace contains:

- `lillis.big.table`: OTU table created with bigger sequence dataset containing airborne bacteria. Swabs are a subset of those samples.
- `swab.map`: metadata mapping file
- `big.streptos.names`: vector of plant chloroplast OTUs - these get removed.
- `big.nbh.names`: vector of OTUs with no GreenGenes hit to bacteria level - these are also excluded from analysis.
- `lillis.big.taxa`: taxonomic assignments from GreenGenes database.

```
load("lillis.RData")
source("functions.R")  #functions for formatting shortcuts.
```

Extract OTU table and remove plant sequences as well as sequences not identified as bacteria.

```
swab.table.tmp <- lillis.big.table[row.names(swab.map), -
which(colnames(lillis.big.table) %in%
    c(big.streptos.names, big.nbh.names))]
swab.taxa <- lillis.big.taxa[-which(row.names(lillis.big.taxa) %in%
c(big.streptos.names,
    big.nbh.names)), ]
```

Some important metrics:

- total sequences: 799616
- total samples: 58
- total number of OTUs in this dataset: 5718
- total number of OTUs in bigger dataset (including air): 10782

Even the most depauperate has >4000 sequences, so rarefy to that level.

```
swab.table <- rrarefy(swab.table.tmp, 4000)
swab.table <- swab.table[, which(apply(swab.table, 2, sum) > 0)]
swab.taxa <- swab.taxa[colnames(swab.table), ]
```

After rarefaction, some important metrics:

- total sequences: 2.32×10^5
- total number of OTUs: 3820

Results:

Are communities indicative of surface type? In other words, do we have 4 distinct communities on these 4 distinct surfaces?

```
swab.can <- vegdist(swab.table, "canberra")
adonis(swab.can ~ swab.map$type)
```

```
##
## Call:
## adonis(formula = swab.can ~ swab.map$type)
##
## Terms added sequentially (first to last)
##
##               Df SumsOfSqs MeanSqs F.Model    R2 Pr(>F)
## swab.map$type  3       1.9   0.635    2.29 0.113  0.001 ***
## Residuals     54      15.0   0.277         0.887
## Total         57      16.9                 1.000
## ---
## Signif. codes:  0 '***' 0.001 '**' 0.01 '*' 0.05 '.' 0.1 ' ' 1
```

Yes. Definitely.

Distance-based Redundancy Analysis (DB-RDA) for discriminant analysis as well as visualization:

```
swab.caps <- capscale(swab.can ~ swab.map$type)
anova(swab.caps)
```

```
## Permutation test for capscale under reduced model
##
## Model: capscale(formula = swab.can ~ swab.map$type)
##          Df  Var    F N.Perm Pr(>F)
## Model     3  1.9 2.29    199  0.005 **
## Residual 54 15.0
## ---
## Signif. codes:  0 '***' 0.001 '**' 0.01 '*' 0.05 '.' 0.1 ' ' 1
```

This test is essentially identical, but with different iterations, so different p-value. Create a version of the DB-RDA that will have individual OTUs weighting samples:

```
swab.caps2 <- capscale(swab.table ~ swab.map$type, distance =
"canberra")
anova(swab.caps2)
```

```
## Permutation test for capscale under reduced model
##
## Model: capscale(formula = swab.table ~ swab.map$type, distance =
"canberra")
##          Df  Var    F N.Perm Pr(>F)
## Model     3  1.9 2.29    199  0.005 **
## Residual 54 15.0
## ---
## Signif. codes:  0 '***' 0.001 '**' 0.01 '*' 0.05 '.' 0.1 ' ' 1
```

```
scaps2.plot <- plot(swab.caps2)
```

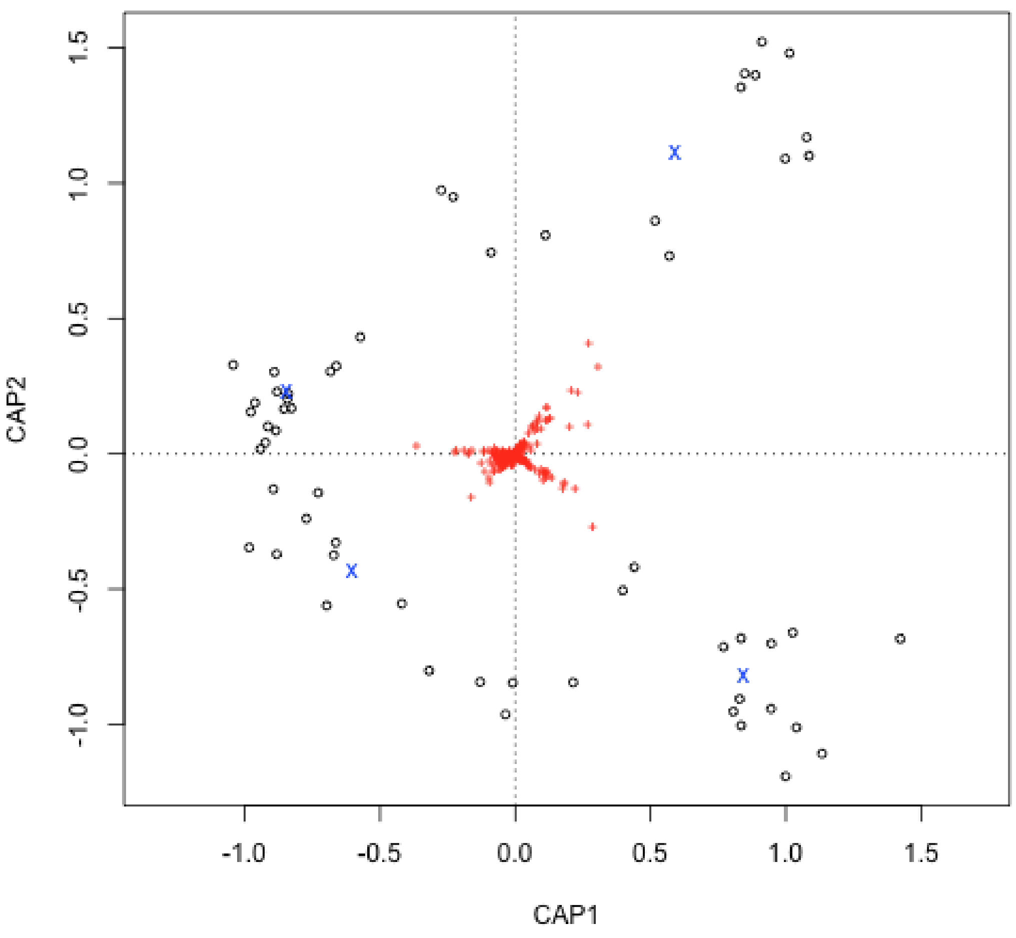

This is all to get a dataframe for each set of indicator taxa sets the edit commands were used to manually change taxa names for plotting.

```
sp.caps <- data.frame(scaps2.plot$species[, c(1, 2)])
chair.caps.all <- sp.caps[which(sp.caps$CAP1 > 0 & sp.caps$CAP2 > 0),
]
desk.caps.all <- sp.caps[which(sp.caps$CAP1 > 0 & sp.caps$CAP2 < 0),
]
wall.caps.all <- sp.caps[which(sp.caps$CAP1 < 0 & sp.caps$CAP2 < 0),
]
floor.caps.all <- sp.caps[which(sp.caps$CAP1 < 0 & sp.caps$CAP2 > 0),
]
```

Function to find extant OTUs

```
hypot <- function(d.f) {
    sqrt.d.f <- sqrt((d.f[, 1]^2) + (d.f[, 2]^2))
    invisible(sqrt.d.f)
}
```

Highlight those OTUs with strongest weights for each of the 4 groups. Started with top 10, then trimmed to significant indicators (from analysis below).

```
hypot.chair <- hypot(chair.caps.all)
chair.tops <- chair.caps.all[rev(order(hypot.chair)), ][1:8, ]

hypot.desk <- hypot(desk.caps.all)
desk.tops <- desk.caps.all[rev(order(hypot.desk)), ][1:5, ]

hypot.wall <- hypot(wall.caps.all)
wall.tops <- wall.caps.all[rev(order(hypot.wall)), ][1:4, ]

hypot.floor <- hypot(floor.caps.all)
floor.tops <- floor.caps.all[rev(order(hypot.floor)), ][1:6, ]
```

Then save species scores for easier plotting.

```
sp.caps <- scores(swab.caps2)$species
```

Put taxon names on the OTUs. This requires the two functions intended to format taxonomy output. The output is a OTU classification dataframe with total abundance in the last column.

```
taxo <- cleanTaxo(makeTaxo(taxo.in = swab.taxa$taxa.names, otu.table
= swab.table,
    split = ";"))
```

```
## Warning: no non-missing arguments to max; returning -Inf Warning:
no
## non-missing arguments to max; returning -Inf Warning: no non-
missing
## arguments to max; returning -Inf Warning: no non-missing arguments
to max;
## returning -Inf Warning: no non-missing arguments to max; returning
-Inf
## Warning: no non-missing arguments to max; returning -Inf
```

```
head(taxo)
```

```
##     kingdom         phylum                class               order
## 1  Bacteria Actinobacteria      Actinobacteria    Actinomycetales
## 7  Bacteria            SR1                   -                   -
## 9  Bacteria Proteobacteria Gammaproteobacteria    Pseudomonadales
## 10 Bacteria Proteobacteria Alphaproteobacteria   Sphingomonadales
## 11 Bacteria  Bacteroidetes     Sphingobacteria Sphingobacteriales
## 12 Bacteria Proteobacteria Alphaproteobacteria        Rhizobiales
##                family           genus abundance
## 1  Corynebacteriaceae Corynebacterium         1
## 7                   -               -       850
## 9       Moraxellaceae   Acinetobacter         1
## 10  Sphingomonadaceae    Sphingomonas      1715
## 11   Flexibacteraceae     Dyadobacter       153
## 12                  -               -        10
```

Put names on the right points.

```
chair.tops$taxa <- taxo[row.names(chair.tops), 6]
desk.tops$taxa <- taxo[row.names(desk.tops), 6]
wall.tops$taxa <- taxo[row.names(wall.tops), 6]
floor.tops$taxa <- taxo[row.names(floor.tops), 6]

chair.tops$pos <- 1
desk.tops$pos <- 1
wall.tops$pos <- 1
floor.tops$pos <- 1

cap.txt <- data.frame(x = c(1.3, 1.1, -0.8, -1), y = c(0.75, -0.2,
-1, 0.75))
```

Check for significance of each indicator pointed out. This uses the `indval` function in the `labdsv` package - it essentially follows the procedure outlined in Dufrene & Legendre (1998). A few steps follow to format `indval` output for downstream analysis.

```
indic <- indval(swab.table, swab.map$type)

ch.ids <- paste("X", row.names(chair.tops), sep = "")
de.ids <- paste("X", row.names(desk.tops), sep = "")
wa.ids <- paste("X", row.names(wall.tops), sep = "")
fl.ids <- paste("X", row.names(floor.tops), sep = "")

chair.tops$indic <- indic$pval[ch.ids]
desk.tops$indic <- indic$pval[de.ids]
wall.tops$indic <- indic$pval[wa.ids]
floor.tops$indic <- indic$pval[fl.ids]

all.tops <- rbind(chair.tops, desk.tops, wall.tops, floor.tops)
all.tops$surface <- factor(c(rep("chair", nrow(chair.tops)),
rep("desk", nrow(desk.tops)),
    rep("wall", nrow(wall.tops)), rep("floor", nrow(floor.tops))))
for (i in 1:nrow(all.tops)) {
    all.tops$chair.ra[i] <- mean(swab.table[swab.map$type == "chair",
row.names(all.tops)[i]]/sum(swab.table[swab.map$type ==
        "chair", ]))
    all.tops$desk.ra[i] <- mean(swab.table[swab.map$type == "desk",
row.names(all.tops)[i]]/sum(swab.table[swab.map$type ==
        "desk", ]))
    all.tops$wall.ra[i] <- mean(swab.table[swab.map$type == "wall",
row.names(all.tops)[i]]/sum(swab.table[swab.map$type ==
        "wall", ]))
    all.tops$floor.ra[i] <- mean(swab.table[swab.map$type == "floor",
row.names(all.tops)[i]]/sum(swab.table[swab.map$type ==
        "floor", ]))
}

all.tops
```

```
##             CAP1      CAP2                  taxa pos indic surface
chair.ra
## 17     0.27276  0.415179          Lactobacillus   1 0.001   chair
0.0024069
## 141    0.30537  0.325324       Corynebacterium   1 0.001   chair
0.0025804
## 639    0.23063  0.233268       Corynebacterium   1 0.001   chair
0.0018406
## 10800  0.20817  0.234748       Corynebacterium   1 0.001   chair
0.0017347
## 216    0.26771  0.110892        Staphylococcus   1 0.014   chair
0.0015344
## 188    0.20078  0.100124        Staphylococcus   1 0.006   chair
0.0011888
## 64     0.11712  0.176516          Lactobacillus   1 0.001   chair
0.0010191
## 151    0.11639  0.176967          Lactobacillus   1 0.001   chair
0.0010485
## 135    0.28550 -0.269799 CandidatusPhytoplasma   1 0.012    desk
0.0002028
## 530    0.22064 -0.125365          Streptococcus   1 0.001    desk
0.0003355
## 1333   0.17556 -0.125379          Streptococcus   1 0.001    desk
0.0001314
## 153    0.18022 -0.111329          Streptococcus   1 0.001    desk
0.0002309
## 1961   0.18254 -0.100863          Brevundimonas   1 0.001    desk
```

```
0.0003214
## 942   -0.16204 -0.153377      Alicyclobacillus   1 0.001    wall
0.0007474
## 314   -0.09518 -0.102071      Alicyclobacillus   1 0.001    wall
0.0004783
## 82    -0.12505 -0.028408          Yonghaparkia   1 0.051    wall
0.0006824
## 21    -0.11186 -0.061582          Sphingomonas   1 0.073    wall
0.0010293
## 174   -0.36570  0.030164            Salmonella   1 0.001   floor
0.0002156
## 1312  -0.22237  0.005107     Chroococcidiopsis   1 0.143   floor
0.0007296
## 1076  -0.21807  0.017430            Roseomonas   1 0.001   floor
0.0001390
## 1203  -0.18912  0.018228     Chroococcidiopsis   1 0.082   floor
0.0006008
## 164   -0.17267  0.004875     Chroococcidiopsis   1 0.077   floor
0.0004464
## 596   -0.17030  0.011427                     -   1 0.001   floor
0.0001071
##          desk.ra   wall.ra  floor.ra
## 17    1.200e-04 1.233e-04 7.908e-05
## 141   7.756e-04 5.267e-04 4.235e-04
## 639   5.778e-04 3.144e-04 3.036e-04
## 10800 4.556e-04 2.711e-04 2.602e-04
## 216   9.978e-04 3.389e-04 3.278e-04
## 188   7.300e-04 1.922e-04 3.023e-04
## 64    4.444e-05 4.889e-05 1.913e-05
## 151   6.333e-05 8.222e-05 3.827e-05
## 135   1.853e-03 3.544e-04 2.577e-04
## 530   1.162e-03 1.367e-04 1.276e-04
## 1333  9.311e-04 7.778e-05 3.954e-05
## 153   9.644e-04 9.556e-05 1.046e-04
## 1961  9.756e-04 1.556e-04 1.173e-04
## 942   9.622e-04 2.127e-03 8.495e-04
## 314   6.633e-04 1.331e-03 5.714e-04
## 82    7.567e-04 1.022e-03 1.254e-03
## 21    1.292e-03 1.403e-03 1.717e-03
## 174   3.256e-04 5.444e-04 2.272e-03
## 1312  4.600e-04 1.304e-03 1.300e-03
## 1076  1.844e-04 3.589e-04 1.323e-03
## 1203  3.489e-04 9.911e-04 1.140e-03
## 164   2.533e-04 8.844e-04 9.120e-04
## 596   1.633e-04 2.778e-04 1.056e-03
```

Then write a table for manual documentation of individual OTUs. This goes into Table 1.

```
write.table(all.tops, file = "surface_indicators.txt", sep = "\t",
quote = FALSE)
```

Put surface samples in context with gut, skin, soil, phyllosphere. First read in `sources.csv`; this is a Class-level OTU table from multiple source environments. The same approach was used in Kembel et al. (2012) to identify potential sources in airborne hospital bacterial assemblages.

```
## source environments
sources.tmp <- read.csv("sourceHabitatsBlastClass.csv")
sources <- sources.tmp[, c(2, 4, 3)]
names(sources) <- c("sample", "species", "abundance")

## prepare surface OTU table
swabClass <- aggregate(t(swab.table), by = list(taxo$class), FUN =
"sum")
row.names(swabClass) <- swabClass[, 1]
swabClass <- swabClass[, -1]
swabClass <- data.frame(t(swabClass/4000))
swabClass <- dematrify(swabClass)

## combine and make longform
sourcesAll.tmp <- rbind(swabClass, sources)
sourcesAll <- matrify(sourcesAll.tmp)

## make mapping factor
sourcesMap <- data.frame(env = rep("skin", nrow(sourcesAll)))
row.names(sourcesMap) <- rn <- row.names(sourcesAll)
sourcesMap$env <- as.character(sourcesMap$env)
sourcesMap$env[grep("Swab", rn)] <- "classroom"
sourcesMap$env[grep("Fcsw", rn)] <- "gut"
sourcesMap$env[grep("phyllosphere", rn)] <- "phyllosphere"
sourcesMap$env[grep("soil", rn)] <- "soil"
sourcesMap$env[grep("water", rn)] <- "water"
sourcesMap$env <- as.factor(sourcesMap$env)

sourcesMap$bg <- "transparent"
sourcesMap$bg[1:58] <- swab.map$bg
```

Then create ordination of these sources combined with surfaces samples.

```
sourcesPCA <- pca(sourcesAll)
plantskinPCA1 <- sourcesPCA$scores[, 1]
```

How much variance was explained by the axes?

```
(sourcesPCA$sdev[c(1:2)]^2)/sum(sourcesPCA$sdev^2)
```

```
## [1] 0.3787 0.2304
```

Plot results

```
sourcesMap$col <- 1
for (i in 1:nlevels(sourcesMap$env)) {
    sourcesMap$col[sourcesMap$env == levels(sourcesMap$env)[i]] <- i
}
plot(sourcesPCA, type = "n")
points(sourcesPCA$scores[1:58, 1:2], pch = 21, bg =
sourcesMap$bg[1:58], cex = 2)
points(sourcesPCA$scores[-c(1:58), 1:2], col = sourcesMap$col[-
c(1:58)], pch = 16)
legend("bottomleft", legend = c("Chair", "Desk", "Wall", "Floor",
levels(sourcesMap$env)[-1]),
    pch = c(rep(21, 4), rep(16, 5)), col = c(1, 1, 1, 1, 2:6), pt.bg
= c("darkslateblue",
        "goldenrod3", "wheat", "chocolate3"))
```

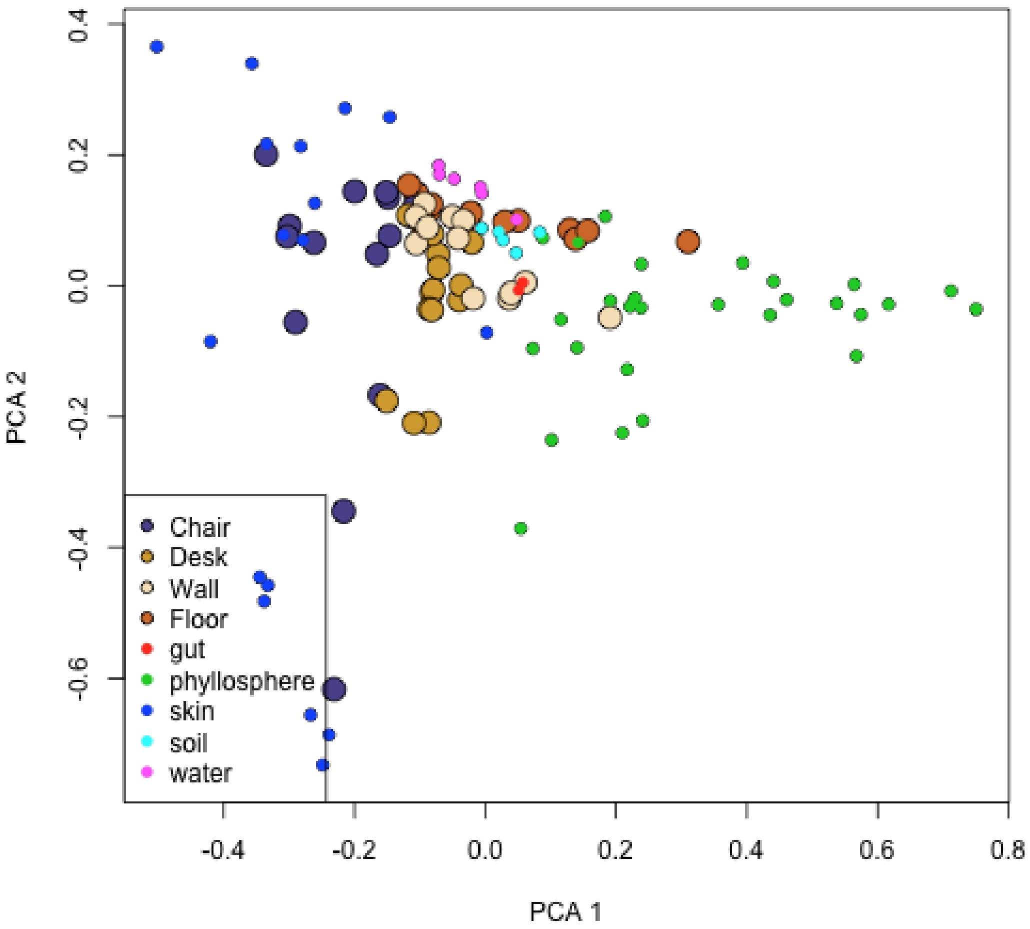


```
boxType <- factor(swab.map$type, levels = c("chair", "desk", "wall",
"floor"))

library(IDPmisc)
```

```
## Loading required package: grid Loading required package: lattice
```

```
par(mar = c(5, 6, 2, 1))
boxplot(swab.map$plantskin ~ boxType, notch = TRUE, col =
c("darkslateblue",
    "goldenrod3", "wheat", "chocolate3"), ylab = "", las = 1, pch =
21, bg = c("wheat",
    "goldenrod3"), pch = 1.5)
```

```
## Warning: some notches went outside hinges ('box'): maybe set
notch=FALSE
```

```
par(xpd = TRUE)
Arrows(-0.2, -0.0078, -0.2, -0.4317, sh.col = "gray20", sh.lwd = 2.5)
Arrows(-0.2, -0.4317, -0.2, -0.0078, sh.col = "gray20", sh.lwd = 2.5)
mtext(c("more like Skin", "more like Plants"), side = 2, at = c(-0.7,
0.3),
    line = 3)
text(c(1, 2, 3, 3.85), c(rep(0.4, 4)), c("a", "b", "bc", "c"), font =
1, cex = 1.4)
```

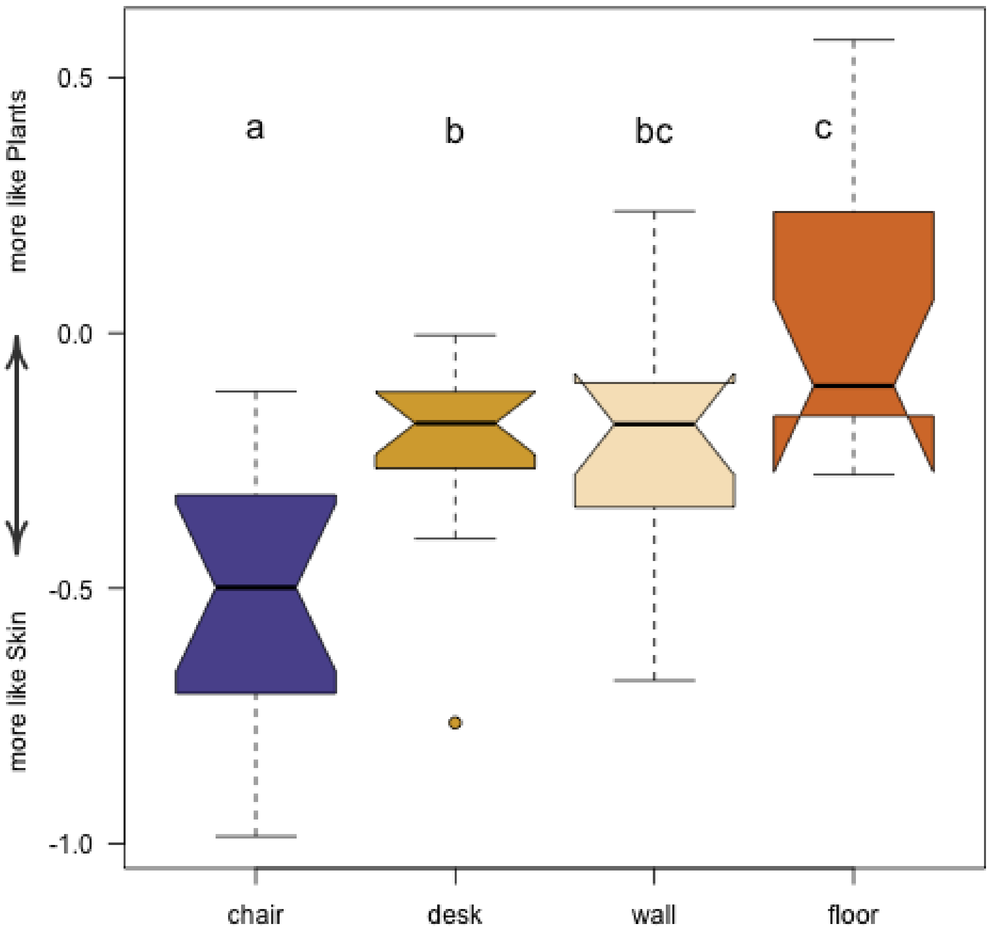


```
TukeyHSD(aov(swab.map$plantskin ~ swab.map$type))
```

```
##   Tukey multiple comparisons of means
##     95% family-wise confidence level
##
## Fit: aov(formula = swab.map$plantskin ~ swab.map$type)
##
## $`swab.map$type`
##                  diff       lwr      upr  p adj
## desk-chair   0.29182  0.06229 0.521342 0.0074
## floor-chair  0.52387  0.29042 0.757322 0.0000
## wall-chair   0.30429  0.07477 0.533818 0.0048
## floor-desk   0.23206  0.00253 0.461581 0.0466
## wall-desk    0.01248 -0.21306 0.238009 0.9989
## wall-floor  -0.21958 -0.44911 0.009946 0.0656
```

This revised version of the figure contains information from the source PCA performed above. First some baggage from previous analysis.

```
library(IDPmisc)
boxType <- factor(swab.map$type, levels = c("chair", "desk", "wall",
"floor"))
boxTypeNum <- rep(1, nrow(swab.map))
boxTypeNum[boxType == "chair"] <- 1
boxTypeNum[boxType == "desk"] <- 2
boxTypeNum[boxType == "wall"] <- 3
boxTypeNum[boxType == "floor"] <- 4

chair.caps <- chair.tops
desk.caps <- desk.tops
wall.caps <- wall.tops
floor.caps <- floor.tops
```

```
layout(matrix(c(1, 2, 3), 1, 3), widths = c(1, 1, 0.75))
par(xpd = FALSE, mar = c(3, 3, 3, 1), las = 0)
plot(swab.caps, type = "none", xaxt = "n", yaxt = "n", xlab = "",
ylab = "")
mtext(c("CAP 1", "CAP 2"), side = c(1, 2), line = c(0.3, 0.1), adj =
c(1, 1))
points(swab.caps, "sites", pch = 21, bg = swab.map$bg, cex = 2)
text(c(cap.txt$x), c(cap.txt$y), c("Chairs", "Desks", "Walls",
"Floors"), cex = 1)
mtext("a", adj = 0, font = 2, cex = 2)
plot(sp.caps, pch = 16, cex = 0.4, col = 8, xlim = c(range(sp.caps)),
ylim = c(range(sp.caps)),
    xaxt = "n", yaxt = "n", xlab = "", ylab = "")
mtext(c("CAP 1", "CAP 2"), side = c(1, 2), line = c(0.3, 0.1), adj =
c(1, 1))
abline(h = 0, v = 0, lty = 3, lwd = 0.7, col = 1)
# rect(-1.5, -1.5, 2, 2)
par(xpd = TRUE)
points(chair.caps$CAP1, chair.caps$CAP2, pch = 21, bg =
"darkslateblue", cex = 1,
    lwd = 0.5)
points(desk.caps$CAP1, desk.caps$CAP2, pch = 21, bg = "goldenrod3",
cex = 1,
    lwd = 0.5)
points(wall.caps$CAP1, wall.caps$CAP2, pch = 21, bg = "wheat", cex =
1, lwd = 0.5)
points(floor.caps$CAP1, floor.caps$CAP2, pch = 21, bg = "chocolate3",
cex = 1,
    lwd = 0.5)
text(chair.caps$CAP1, chair.caps$CAP2, as.character(chair.caps$taxa),
cex = 0.7,
    pos = chair.caps$pos)
text(desk.caps$CAP1, desk.caps$CAP2, as.character(desk.caps$taxa),
cex = 0.7,
    pos = desk.caps$pos)
text(wall.caps$CAP1, wall.caps$CAP2, as.character(wall.caps$taxa),
cex = 0.7,
    pos = wall.caps$pos)
text(floor.caps$CAP1, floor.caps$CAP2, as.character(floor.caps$taxa),
cex = 0.7,
    pos = floor.caps$pos)
mtext("b", adj = 0, font = 2, cex = 2)
par(mar = c(4, 5, 3, 1))
boxplot(swab.map$plantskin ~ boxType, notch = TRUE, col =
c("darkslateblue",
    "goldenrod3", "wheat", "chocolate3"), ylab = "", las = 1, pch =
21, cex = 0,
    bg = c("wheat", "goldenrod3"))
```

```
## Warning: some notches went outside hinges ('box'): maybe set
notch=FALSE
```

```
points(swab.map$plantskin ~ jitter(boxTypeNum, 0.7), pch = 21, bg =
swab.map$bg)
par(xpd = TRUE)
Arrows(-0.55, 0.2, -0.55, -0.7, sh.col = "gray40", sh.lwd = 1.5,
width = 2.5,
    size = 0.5)
Arrows(-0.55, -0.7, -0.55, 0.2, sh.col = "gray40", sh.lwd = 1.5,
width = 2.5,
    size = 0.5)
mtext("Source PCA Score", side = 2, at = -0.25, line = 3.1, col =
"gray30",
    cex = 0.8)
mtext(c("Skin", "Plants"), side = 2, at = c(-0.85, 0.4), line = 2.5)
text(c(1, 2, 3, 3.85), c(rep(0.4, 4)), c("a", "b", "bc", "c"), font =
1, cex = 1.4)
mtext("c", adj = 0, font = 2, cex = 2)
```

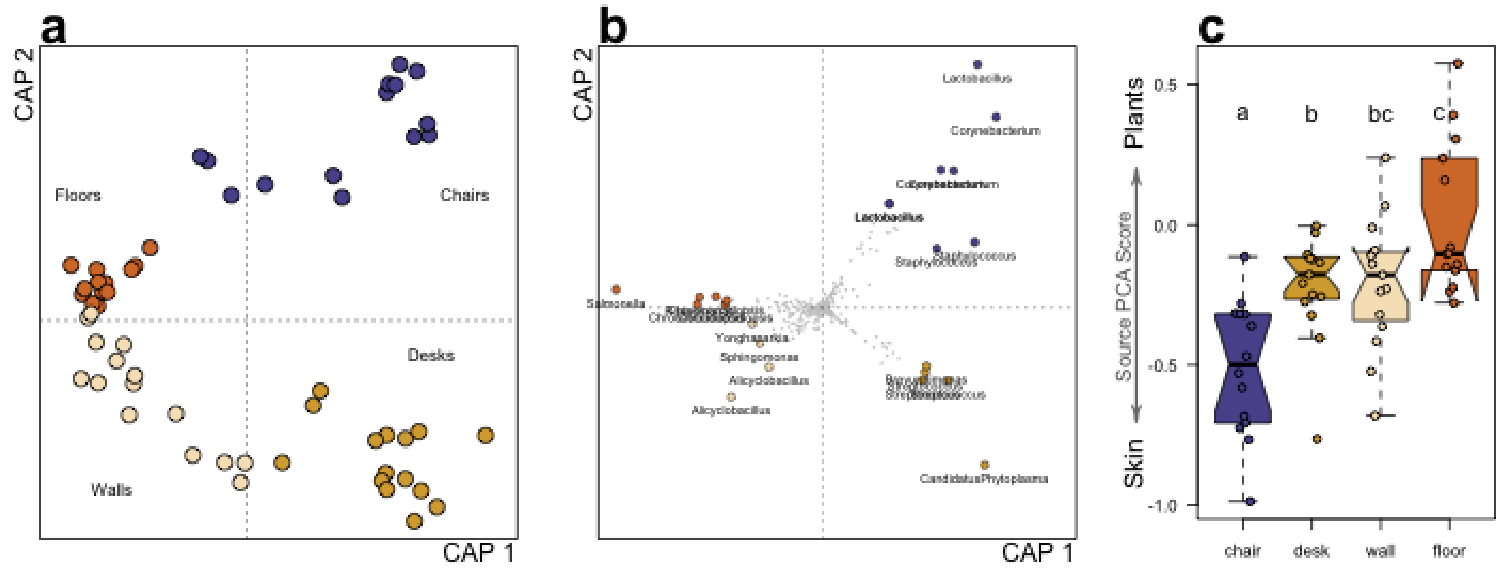

Create subsets of data for Mantel tests. Ie, test for geographic location within the room.

```
st.c <- swab.table[swab.map$type == "chair", ]
st.f <- swab.table[swab.map$type == "floor", ]
st.d <- swab.table[swab.map$type == "desk", ]
st.w <- swab.table[swab.map$type == "wall", ]

sm.c <- swab.map[swab.map$type == "chair", ]
sm.f <- swab.map[swab.map$type == "floor", ]
sm.d <- swab.map[swab.map$type == "desk", ]
sm.w <- swab.map[swab.map$type == "wall", ]

mantel(vegdist(st.c, "canberra"), vegdist(data.frame(sm.c$xcor,
sm.c$ycor),
    "euclid"))
```

```
##
## Mantel statistic based on Pearson's product-moment correlation
##
## Call:
## mantel(xdis = vegdist(st.c, "canberra"), ydis =
vegdist(data.frame(sm.c$xcor,      sm.c$ycor), "euclid"))
##
## Mantel statistic r: 0.0109
##       Significance: 0.45
##
## Upper quantiles of permutations (null model):
##   90%   95% 97.5%   99%
## 0.178 0.218 0.278 0.340
##
## Based on 999 permutations
```

```
mantel(vegdist(st.f, "canberra"), vegdist(data.frame(sm.f$xcor,
sm.f$ycor),
    "euclid"))
```

```
##
## Mantel statistic based on Pearson's product-moment correlation
##
## Call:
## mantel(xdis = vegdist(st.f, "canberra"), ydis =
vegdist(data.frame(sm.f$xcor,      sm.f$ycor), "euclid"))
##
## Mantel statistic r: 0.0437
##       Significance: 0.39
##
## Upper quantiles of permutations (null model):
##   90%   95% 97.5%   99%
## 0.194 0.232 0.287 0.346
##
## Based on 999 permutations
```

```
mantel(vegdist(st.d, "canberra"), vegdist(data.frame(sm.d$xcor,
sm.d$ycor),
    "euclid"))
```

```
##
## Mantel statistic based on Pearson's product-moment correlation
##
## Call:
## mantel(xdis = vegdist(st.d, "canberra"), ydis =
vegdist(data.frame(sm.d$xcor,      sm.d$ycor), "euclid"))
##
## Mantel statistic r: -0.0441
##       Significance: 0.61
##
## Upper quantiles of permutations (null model):
##   90%   95% 97.5%   99%
## 0.148 0.198 0.234 0.302
##
## Based on 999 permutations
```

```
mantel(vegdist(st.w, "canberra"), vegdist(data.frame(sm.w$xcor,
sm.w$ycor),
    "euclid"))
```

```
##
## Mantel statistic based on Pearson's product-moment correlation
##
## Call:
## mantel(xdis = vegdist(st.w, "canberra"), ydis =
vegdist(data.frame(sm.w$xcor,      sm.w$ycor), "euclid"))
##
## Mantel statistic r: -0.06
##       Significance: 0.79
##
## Upper quantiles of permutations (null model):
##    90%    95%  97.5%    99%
## 0.0982 0.1535 0.2060 0.2721
##
## Based on 999 permutations
```

```
mantel(vegdist(swab.table, "canberra"),
vegdist(data.frame(swab.map$xcor, swab.map$ycor),
    "euclid"))
```

```
##
## Mantel statistic based on Pearson's product-moment correlation
##
## Call:
## mantel(xdis = vegdist(swab.table, "canberra"), ydis =
vegdist(data.frame(swab.map$xcor,      swab.map$ycor), "euclid"))
##
## Mantel statistic r: -0.00269
##       Significance: 0.52
##
## Upper quantiles of permutations (null model):
##    90%    95%  97.5%    99%
## 0.0677 0.0864 0.1023 0.1163
##
## Based on 999 permutations
```

```
plot(vegdist(swab.table, "canberra"),
vegdist(data.frame(swab.map$xcor, swab.map$ycor),
    "euclid"))
```

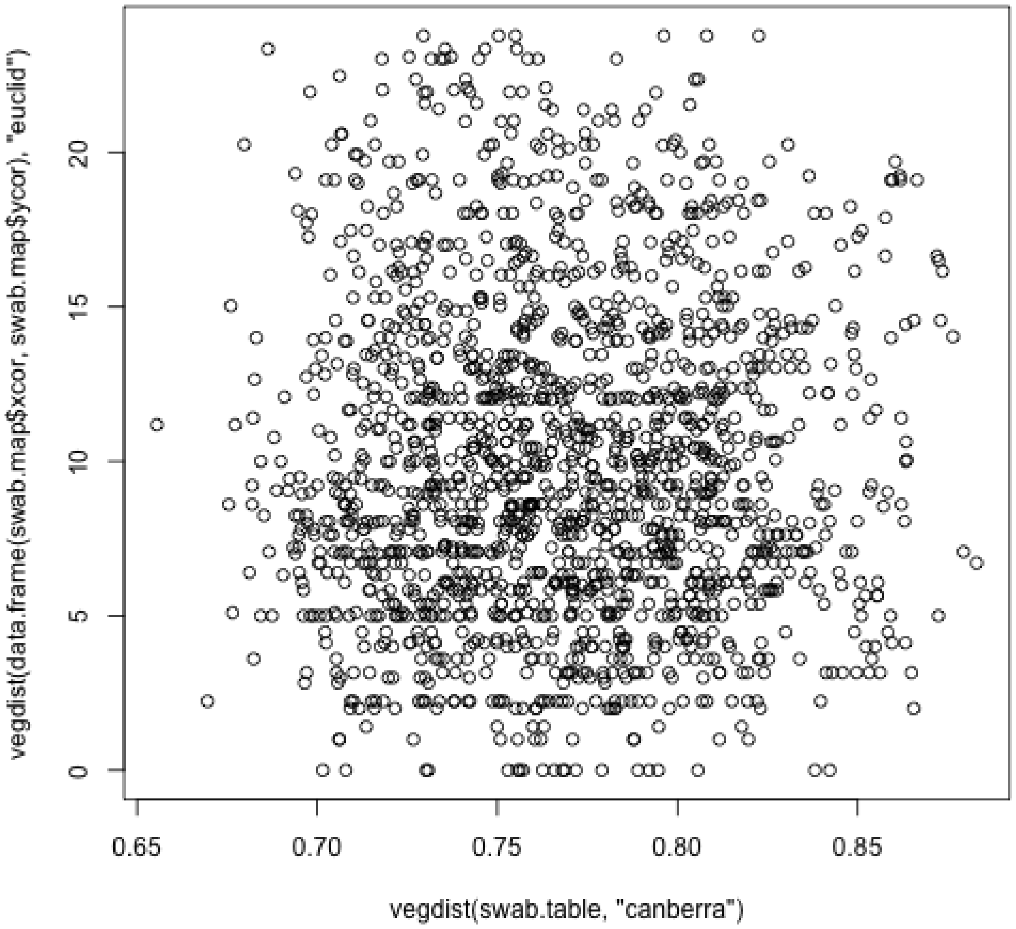


```
cor.test(vegdist(swab.table, "canberra"),
vegdist(data.frame(swab.map$xcor,
    swab.map$ycor), "euclid"))
```

```
##
##  Pearson's product-moment correlation
##
## data:  vegdist(swab.table, "canberra") and
vegdist(data.frame(swab.map$xcor, swab.map$ycor), "euclid")
## t = -0.1092, df = 1651, p-value = 0.913
## alternative hypothesis: true correlation is not equal to 0
## 95 percent confidence interval:
##  -0.05089  0.04553
## sample estimates:
##       cor
## -0.002688
```

```
# Mantel statistic r: 0.01047 Significance: 0.399
```

This is never significant, which indicates that surface type makes more difference than location around the room (eg, proximity to door, window, …).

And just to check, does it matter whether the walls were sampled high or low?

```
sm.w$location2 <- factor(sm.w$location2)
adonis(vegdist(st.w, "canberra") ~ sm.w$location2)
```

```
##
## Call:
## adonis(formula = vegdist(st.w, "canberra") ~ sm.w$location2)
##
## Terms added sequentially (first to last)
##
##                Df SumsOfSqs MeanSqs F.Model    R2 Pr(>F)
## sm.w$location2  1      0.30   0.301    1.06 0.075   0.24
## Residuals      13      3.69   0.284         0.925
## Total          14      3.99                 1.000
```

Nope.

Make stacked barchart of OTU relative abundances. This takes the `taxo` table created above.

```
t.phylum <- aggregate(t(swab.table), by = list(taxo$phylum), sum)
t.class <- aggregate(t(swab.table), by = list(taxo$class), sum)
t.order <- aggregate(t(swab.table), by = list(taxo$order), sum)
```

Then order everything for visual greatness.

```
#### phylum  1% cutoff
row.names(t.phylum) <- t.phylum$Group.1
t.phylum <- t.phylum[, -1]
t.phylum <- t.phylum[order(rowSums(t.phylum)), order(swab.map$type)]
nrows <- nrow(t.phylum)
nshow <- 7
nkeep <- nrows-nshow+1
t.phylum <- rbind(colSums(t.phylum[1:nkeep-1, ]),
t.phylum[nkeep:nrows, ])
cols.8 <- c('#D73027', '#FC8D59', '#FEE090', '#FFFFBF', '#E0F3F8',
'#91BFDB', '#9475B4', '#969A97')

# class  1% cutoff
row.names(t.class) <- t.class$Group.1
t.class <- t.class[, -1]
t.class <- t.class[order(rowSums(t.class)), order(swab.map$type)]
nrows <- nrow(t.class)
nshow <- 10
nkeep <- nrows-nshow+1
t.class <- rbind(colSums(t.class[1:nkeep-1, ]), t.class[nkeep:nrows,
])
t.class <- cbind(t.class[, 1:14][, rev(order(t.phylum[8, 1:14]))],
                  t.class[, 15:29][, rev(order(t.phylum[8, 15:29]))],
                  t.class[, 30:43][, rev(order(t.phylum[8, 30:43]))],
                  t.class[, 44:58][, rev(order(t.phylum[8, 44:58]))]
)
cols.11 <- c('#A50026', '#D73027', '#F46D43', '#FDAE61', '#FEE090',
             '#E0F3F8', '#ABD9E9', '#74ADD1', '#4575B4', '#313695',
'#969A97')

# order
row.names(t.order) <- t.order$Group.1
t.order <- t.order[, -1]
t.order <- t.order[order(rowSums(t.order)), order(swab.map$type)]
nrows <- nrow(t.order)
nshow <- 10
nkeep <- nrows-nshow+1
t.order <- rbind(colSums(t.order[1:nkeep-1, ]), t.order[nkeep:nrows,
])
t.order <- cbind(t.order[, 1:14][, rev(order(t.phylum[8, 1:14]))],
                  t.order[, 15:29][, rev(order(t.phylum[8, 15:29]))],
                  t.order[, 30:43][, rev(order(t.phylum[8, 30:43]))],
                  t.order[, 44:58][, rev(order(t.phylum[8, 44:58]))]
)
cols.11 <- c('#A50026', '#D73027', '#F46D43', '#FDAE61', '#FEE090',
             '#E0F3F8', '#ABD9E9', '#74ADD1', '#4575B4', '#313695',
'#969A97')
# create index for bar mid-points.
bar <- barplot(as.matrix(t.order/4000), col=rev(cols.11))
```

```
####  this had to be run las to maintain phylum order in other charts
t.phylum <- cbind(t.phylum[, 1:14][, rev(order(t.phylum[8, 1:14]))],
              t.phylum[, 15:29][, rev(order(t.phylum[8, 15:29]))],
                  t.phylum[, 30:43][, rev(order(t.phylum[8,
30:43]))],
                  t.phylum[, 44:58][, rev(order(t.phylum[8, 44:58]))]
)


ph.names <- row.names(t.phylum)
ph.names[1] <- 'other (<1%)'
cl.names <- row.names(t.class)
cl.names[1] <- 'other (<1%)'
or.names <- row.names(t.order)
or.names[1] <- 'other (<4%)'
```

Figure 3. Taxonomy bar chart.

```
layout(matrix(c(1, 2, 3, 7, 1, 2, 3, 7, 4, 5, 6, 7), 4, 3), heights =
c(1, 1,
    1, 0.1))
par(mar = c(1, 4, 2, 1))
barplot(as.matrix(t.phylum/4000), col = rev(cols.8), border =
rev(cols.8), xaxt = "n",
    las = 1)
mtext("Phylum", font = 2, col = "gray20")
segments(c(mean(bar[c(14, 15)]), mean(bar[c(29, 30)]), mean(bar[c(43,
44)])),
    c(0, 0, 0), c(mean(bar[c(14, 15)]), mean(bar[c(29, 30)]),
mean(bar[c(43,
        44)])), c(1, 1, 1))

mtext(c("chairs", "desks", "floors", "walls"), side = 1, at =
c(mean(bar[c(1,
    14)]), mean(bar[c(15, 29)]), mean(bar[c(30, 43)]), mean(bar[c(44,
58)])),
    font = 2, col = "gray20")
barplot(as.matrix(t.class/4000), col = rev(cols.11), border =
rev(cols.11),
    xaxt = "n", las = 1)
mtext("Class", font = 2, col = "gray20")
segments(c(mean(bar[c(14, 15)]), mean(bar[c(29, 30)]), mean(bar[c(43,
44)])),
    c(0, 0, 0), c(mean(bar[c(14, 15)]), mean(bar[c(29, 30)]),
mean(bar[c(43,
        44)])), c(1, 1, 1))
mtext(c("chairs", "desks", "floors", "walls"), side = 1, at =
c(mean(bar[c(1,
    14)]), mean(bar[c(15, 29)]), mean(bar[c(30, 43)]), mean(bar[c(44,
58)])),
    font = 2, col = "gray20")
barplot(as.matrix(t.order/4000), col = rev(cols.11), border =
rev(cols.11),
    xaxt = "n", las = 1)
mtext("Order", font = 2, col = "gray20")
segments(c(mean(bar[c(14, 15)]), mean(bar[c(29, 30)]), mean(bar[c(43,
44)])),
    c(0, 0, 0), c(mean(bar[c(14, 15)]), mean(bar[c(29, 30)]),
mean(bar[c(43,
```

```
        44)])), c(1, 1, 1))
par(xpd = TRUE)
mtext(c("chairs", "desks", "floors", "walls"), side = 1, at =
c(mean(bar[c(1,
    14)]), mean(bar[c(15, 29)]), mean(bar[c(30, 43)]), mean(bar[c(44,
58)])),
    font = 2, col = "gray20")
par(mar = c(0, 0, 0, 0))
plot(1, 1, type = "n", bty = "n", xlab = "", ylab = "", xaxt = "n",
yaxt = "n")
legend("left", legend = c(rev(ph.names)), pt.bg = cols.8, col =
cols.8, bty = "n",
    pch = 22, cex = 1.5, pt.cex = 2.5)
plot(1, 1, type = "n", bty = "n", xlab = "", ylab = "", xaxt = "n",
yaxt = "n")
legend("left", legend = c(rev(cl.names)), pt.bg = cols.11, col =
cols.11, bty = "n",
    pch = 22, cex = 1.5, pt.cex = 2.5)
plot(1, 1, type = "n", bty = "n", xlab = "", ylab = "", xaxt = "n",
yaxt = "n")
legend("left", legend = c(rev(or.names)), pt.bg = cols.11, col =
cols.11, bty = "n",
    pch = 22, cex = 1.5, pt.cex = 2.5)
```

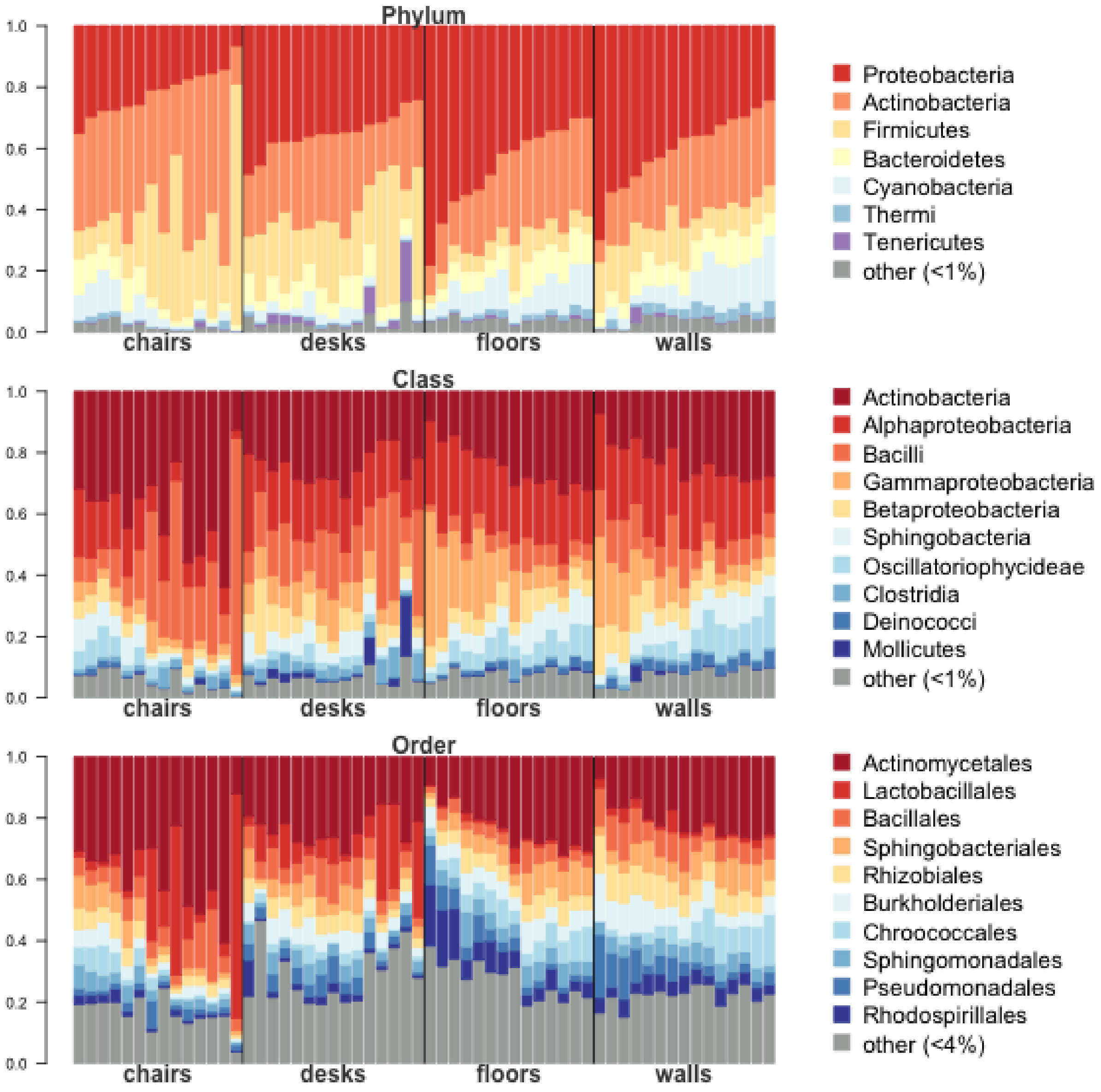

Supplement: Additional file 5 — Full account of statistical analysis performed in R. [file 2049-2618-2-7-S5.pdf]
